# Early HAART Initiation May Not Reduce Actual Reproduction Number and Prevalence of MSM Infection: Perspectives from Coupled within- and between-Host Modelling Studies of Chinese MSM Populations

**DOI:** 10.1371/journal.pone.0150513

**Published:** 2016-03-01

**Authors:** Xiaodan Sun, Yanni Xiao, Sanyi Tang, Zhihang Peng, Jianhong Wu, Ning Wang

**Affiliations:** 1 Department of Applied Mathematics, Xi’an Jiaotong University, Xi’an, Shaanxi, China; 2 College of Mathematics and Information Science, Shaanxi Normal University, Xi’an, Shaanxi, China; 3 School of Public Health, Nanjing Medical University, Nanjing, Jiangsu, China; 4 Laboratory for Industrial and Applied Mathematics, Centre for Disease Modelling, York Institute for Health Research, York University, Toronto, ON, Canada; 5 National Center for AIDS/STD Prevention and Control, Chinese Center for Disease Control and Prevention, Beijing, China; Shanxi University, CHINA

## Abstract

Having a thorough understanding of the infectivity of HIV, time of initiating treatment and emergence of drug resistant virus variants is crucial in mitigating HIV infection. There are many challenges to evaluating the long-term effect of the Highly Active Antiretroviral Therapy (HAART) on disease transmission at the population level. We proposed an individual based model by coupling within-host dynamics and between-host dynamics and conduct stochastic simulation in the group of men who have sex with men (MSM). The mean actual reproduction number is estimated to be 3.6320 (95% confidence interval: [3.46, 3.80]) for MSM group without treatment. Stochastic simulations show that given relatively high (low) level of drug efficacy after emergence of drug resistant variants, early initiation of treatment leads to a less (greater) actual reproduction number, lower (higher) prevalence and less (more) incidences, compared to late initiation of treatment. This implies early initiation of HAART may not always lower the actual reproduction number and prevalence of infection, depending on the level of treatment efficacy after emergence of drug resistant virus variants, frequency of high-risk behaviors and etc. This finding strongly suggests early initiation of HAART should be implemented with great care especially in the settings where the effective drugs are limited. Coupling within-host dynamics with between-host dynamics can provide critical information about impact of HAART on disease transmission and thus help to assist treatment strategy design and HIV/AIDS prevention and control.

## Introduction

Viral loads may be lowered to undetectable levels when HIV patients are under the Highly Active Antiretroviral Therapy (HAART), reducing infectivity [1–5], slowing down progression to AIDS and improving the life quality of HIV patients. The success of HAART has led to suggestion that all HIV-infected individuals should receive HAART (see [6, 7] and references herein).

Patients with early HAART were reported to survive much longer [8], showing personal health benefit from early HAART [9]. In 2009, Granich et al. [10] developed a mathematical model and analyses to show that earlier (immediate) antiviral therapy for people newly diagnosed with HIV is more effective for reducing the infection rate and death rate. Luo et al. [11] developed a mathematical model, ignoring the occurrence of drug resistant variants, to examine potential effects of test-and-treat on HIV epidemic among men who have sex with men (MSM) in China. This study concluded that the test-and-treat policy would lead to decline of the total number of HIV new infections over the period during 2013 and 2022. On the other hand, the work [12, 13] suggested that early therapy should not be implemented in resource-limited settings as early treatment in these settings inevitably induces early occurrence of drug resistance due to drug non-adherence and side-effect resulting in declining treatment efficacy. The studies [14, 15] also reported that HAART did not prevent HIV transmission in certain MSM and IDU groups, and there are other important ethical challenges related to early treatment as pointed out by Sugarman et al. [16]. All of these point out the importance of further in-depth examination of issues relevant to the feasibility and implication of early HAART among particular groups in resource limited areas/countries.

Here we address these issues using reported data from a few Chinese MSM studies. We examine the impact of key HAART specifics including time of HAART initiation, time of emergence of drug resistant virus variants and treatment efficacy on HIV new infection in order to determine whether and when early treatment policy should be recommended for MSM populations in mainland China. We conduct this impact analysis using individual based simulations of a novel within-host viral dynamics model coupled with between-host transmission dynamics model.

There are several studies on coupling together within- and between-host dynamics [17–23]. Some studies assumed within-host dynamics is at its endemic state throughout the course of infection [19], while others only described the mean level of disease progression in vivo of different individuals [21–23] or only described the primary and asymptomatic stages of the HIV progression [24]. Due to the (individual) variability of viral loads and the relatively small MSM population, we propose novel individual based models (IBMs) and simulations to couple within- and between-host dynamics. We begin with a modified classic viral dynamics model incorporating the Weibull function (to reflect the temporal variability of infectivity and viral production rate), and implement between-host transmission dynamics stochastic simulations using the viral dynamics model produced viral loads of infected individuals as inputs. We then parameterize the coupled model system using a few Chinese MSM studies to provide critical information, at both the host and population levels, to inform early HAART outcomes under different scenarios of drug resistance, drug efficacy, and drug adherence. We also use simulations to quantify the contribution at different progression stages to the HIV new infection, and to identify optimal treatment regimes and intervention strategies.

## Materials and Methods

### Within-host viral dynamics Model

We adopt the classic HIV viral dynamics model

$$\begin{array}{c} \left\{ \begin{array}{ccc} \frac{dT}{dt} & = & s-dT-k(t)VT,\\ \frac{dT^{*}}{dt} & = & k\left(t\right)VT-\delta T^{*},\\ \frac{dV}{dt} & = & \lambda \left(t\right)T^{*}-cV, \end{array}\right. \end{array}$$ (1)

where *T*, T* and *V* are the concentrations of uninfected target T cells, productively infected cells and free virus, with the recruitment rate *s* of uninfected CD4+ T cells, the death rates *d* and *δ* for uninfected and infected cells, and free virus clearance rate *c* [25, 26]. To characterize the temporal variation of the infection rate *k*(*t*) and the viral production rate *λ*(*t*), we introduce the Weibull function

$$\begin{array}{c} W\left(t,T_{m},\beta ,\alpha \right)=1-exp[-{\left(\frac{T_{m}-t}{\beta }\right)}^{\alpha }],\;t<T_{m}, \end{array}$$

parameterized by *α*, *β* and *T*_*m*_, the shape, scale and location parameters [27]. Here, Weibull function, widely used to depict the ‘bathtub curve’ which is similar to the trend of development of vial loads within a host, is chosen so that the model can fully describe the whole progression of HIV disease, especially a sudden rise in viral load during AIDS stage. Thus, the infection rate as well as the viral production rate are flexible functions of time t. In particular, with *T*_*m*_ interpreted as the maximum life span of the patient after infection, we have

$$\begin{array}{c} k\left(t\right)=\frac{k}{W(T_{m},\beta_{k},\alpha_{k})},\;\lambda \left(t\right)=\frac{\lambda }{W(T_{m},\beta_{\lambda },\alpha_{\lambda })},\;\;\;t<T_{m}, \end{array}$$ (2)

for appropriate parameters *β*_*k*_, *α*_*k*_ and *β*_*λ*_, *α*_*λ*_. In this formulation, when the location parameter *T*_*m*_ tends to infinity, Eq (1) reduces to the classical HIV viral dynamics model. The detailed definitions and baseline parameter values are listed in ***Table A in*** S2 File.

Our model can predict the entire HIV disease progression in vivo perfectly (***Figure A in*** S1 File). By varying the shape, scale and location parameters for *k*(*t*) and *λ*(*t*), we observe different patterns of HIV progression in vivo (***Figure B in*** S1 File). In particular, we observe that the scale parameter (*β*_*k*_) determines the rate of progression to AIDS stage, the shape parameter (*α*_*k*_) determines the viral loads in the asymptomatic stage and the location parameter (*T*_*m*_) determines the survival time. The results of 1000 simulations with shape, scale and location parameters randomly chosen according to rules outlined in ***Table A in*** S2 File are shown in ***Figure C in*** S1 File, and these results show that the maximal viral loads in the primary stage can reach 5 × 10^5^ per *μL*, and the mean value of viral loads varies from 1.1 × 10^5^ to 2 × 10^5^ copies/*μL* [1] at the AIDS stage. We also generate the frequencies of durations of the primary stage, asymptotical stage and AIDS stage (***Figure D in*** S1 File), from which we observe that the primary, asymptomatic and AIDS stage respectively lasts for 2.5–3.5 months, 6 to 10 years and 2 to 3.5 years and this is in good agreement with Zhou et al. [28].

Once HAART is initiated, the disease progression will be changed and the life span may be extended. Denote by *T*_*t*_ the HARRT initiation time (the time since infection when HAART is initiated), this time is determined by the base line CD4 level *B*_*CD*4_ (the level of CD4 cell counts when the HAART is initiated, for example, *B*_*CD*4_ = 350, or *B*_*CD*4_ = 500). The extended life span is denied by *τ*(*t*) (see calculation below) so the location parameter of *k*(*t*) and *λ*(*t*) changes to *T*_*m*_+*τ*(*t*). This change of the disease progression and life span will be further altered after the emergence of drug-resistant virus. To describe these changes, we modify the corresponding infection rate and viral reproduction rate, (*k*^*b*^(*t*), *λ*^*b*^(*t*)) and (*k*^*a*^(*t*), *λ*^*a*^(*t*)), as follows [29]

$$\begin{array}{c} k^{b}\left(t\right)=\frac{k^{b}}{W(T_{m}+\tau \left(t\right),\beta_{k}^{b},\beta_{k}^{b})},\;\lambda^{b}\left(t\right)=\frac{\lambda^{b}}{W(T_{m}+\tau \left(t\right),\beta_{\lambda }^{b},\beta_{\lambda }^{b})},\;t<T_{r}, \end{array}$$ (3)

and

$$\begin{array}{c} k^{a}\left(t\right)=\frac{k^{a}}{W(T_{m}+\tau \left(t\right),\beta_{k}^{a},\beta_{k}^{a})},\;\lambda^{a}\left(t\right)=\frac{\lambda^{a}}{W(T_{m}+\tau \left(t\right),\beta_{\lambda }^{a},\beta_{\lambda }^{a})},\;T_{r}\leq t<T_{s}. \end{array}$$ (4)

where *T*_*r*_ is time of emergence of drug resistant virus variants, which will be treated as a stochastic variable. In what follows, we introduce *r*_*t*_ so that *T*_*r*_ = *T*_*t*_+*r*_*t*_, so *r*_*t*_ measures the rate drug resistance develops. For simplification, we write *k*^*b*^ = *l*_1_ *k*, *λ*^*b*^ = *l*_1_ *λ* and *k*^*a*^ = *l*_2_ *k*, *λ*^*a*^ = *l*_2_ *λ*, $\beta_{k}^{b}=k_{1}\beta_{k},\beta_{k}^{a}=k_{2}\beta_{k}$, where *l*_1_, *l*_2_, *k*_1_ and *k*_2_ are positive constants.

*τ*(*t*) is calculated as follows:

$$\begin{array}{c} \tau \left(t\right)=\{\begin{array}{c} \frac{\tau_{m}\eta^{b}\left(t-T_{t}\right)}{\tau_{50}^{b}+\eta^{b}\left(t-T_{t}\right)},\;\;T_{t}\leq t<T_{r},\\ \frac{\tau_{m}\eta^{a}\left(t-T_{t}\right)}{\tau_{50}^{b}+\eta^{a}\left(t-T_{t}\right)}+\tau_{T_{r}},\;\;t\geq T_{r}. \end{array} \end{array}$$ (5)

$\eta^{b},\tau_{50}^{b}$ and $\eta^{a},\tau_{50}^{a}$ denote the drug efficacy and sensitivity before and after emergence of drug resistant variants, respectively. It is assumed that $\tau_{50}^{a}=r\tau_{50}^{b}$ with *r* ≥ 1 for simplicity. *τ*_*m*_ denotes the maximum life expectancy since initiating HAART. Constant *τ*_*T*_*r*__ can be chosen such that the function *τ*(*t*) is continuous at *T*_*r*_, i.e.

$$\begin{array}{c} \tau_{T_{r}}=\tau_{m}[\frac{\eta^{b}\left(T_{r}-T_{t}\right)}{\tau_{50}^{b}+\eta^{b}\left(T_{r}-T_{t}\right)}-\frac{\eta^{a}\left(T_{r}-T_{t}\right)}{\tau_{50}^{a}+\eta^{a}\left(T_{r}-T_{t}\right)}]. \end{array}$$

Obviously, function *τ*(*t*) = 0 in the absence of HAART.

Our new model allows us to examine how the baseline CD4 cell counts *B*_*CD*4_ impacts the prolonged life expectance. ***Figure E a-b in*** S1 File shows that life span increases as the baseline CD4 cell count increases when other parameters are fixed. Meanwhile, ***Figure E c-d in*** S1 File shows that smaller *l*_1_ leads to longer life span; while ***Figure E e-f in*** S1 File shows people with larger *k*_1_ and *k*_2_ progress faster to the AIDS stage. Naturally, stronger drug sensitivity(small *τ*_50_) leads to larger life span (***Figure F in*** S1 File), and higher drug efficacy (***Figure G a-d in*** S1 File) as well as late emergence of drug resistant variants(***Figure G e-f in*** S1 File) results in longer life span.

### Between-host transmission model

The coupling with transmission at the population is based on the aforementioned viral dynamics model. We consider a population with individuals which are indicated by subindices. The transmission probability *β*(*v*_*i*_) is an increasing function of the viral load, where *v*_*i*_ denotes the viral load in the *i*th infected individual. We shall use the function proposed by Wilson et.al [4], in which each ten-fold increment in viral load is associated with a 2.45-fold increment increase in the risk of HIV transmission per sexual contact, that is, *β*_1_ = 2.45^log_10_(*V*_1_/*V*_0_)^ *β*_0_, where *β*_0_ is the transmission probability per contact for an HIV infected individual with baseline viral load *V*_0_, and *β*_1_ is the transmission probability when the viral load is *V*_1_, whether above or below the baseline.

For the between-host model, we assume individuals enter the high-risk group (*S*(*t*)) at a constant rate *U* and exit at rate *μ*. Let *I*(*t*) be the number of HIV infected individuals at time *t*. Suppose the infected individual was infected at time $t_{i}^{0}$, then the viral load of the *i*th infected individual at time *t* is $v_{i}\left(t-t_{i}^{0}\right)$, associated with his infection age $t-t_{i}^{0}$ and determined by the within-host viral dynamics model described earlier.

Let *N*(*t*) be the total number of population, i.e. *N*(*t*) = *S*(*t*)+*I*(*t*), then the incidence rate induced by the *i*th infected individual is

$$\begin{array}{c} \beta \left(v_{i}\left(t-t_{i}^{0}\right)\right)c\left(t-t_{i}^{0}\right)\left(1-\eta^{c}p\right)S\left(t\right)/N\left(t\right), \end{array}$$

where *p* is the proportion of condom use and *η*^*c*^ is the condom efficacy, $c(t-t_{i}^{0})$ is a piecewise function which describes the frequency of high-risk behaviors, and is given by

$$\begin{array}{c} c\left(t-t_{i}^{0}\right)=\{\begin{array}{c} c_{1},\;\;\text{Primary}\;\text{stage};\\ c_{2},\;\;\text{Asymptomatic}\;\text{stage};\\ c_{3},\;\;\text{AIDS}\;\text{stage}. \end{array} \end{array}$$ (6)

### Stochastic simulation

When an infected individual is introduced to a fully susceptible population, secondary infections will be produced and these will generate further infections in the populations. We implement the stochastic simulations using the *τ*-leap method, where viral loads in every time step Δ*t* is regard as a constant (assuming of course the sufficiently small time step). A simulation is terminated with a termination condition, which in our case is when *M* infected individuals are dead. For each newly infected individual, parameters related to the viral infection rate and production rate in vivo are generated according to the corresponding probability distributions, as ***Table A in*** S2 File shows. Then, viral loads at any infection age are governed by our within-host dynamics model. We approximate the number of new infections by the *i*th infected individual at every time step Δ*t* = 1 day, denoted by *NI*_*i*_, according to

$$\begin{array}{c} NI_{i}∼poisson\left(\beta \left(v_{i}\left(t-t_{i}^{0}\right)\right)c\left(t-t_{i}^{0}\right)\left(1-\eta^{c}p\right)S/N\Delta t\right). \end{array}$$

Similarly, newly recruited susceptibles and individuals who leave the high-risk population at every time step Δ*t* = 1 day are simulated by

$$\begin{array}{ccc} Recruit & ∼ & poisson(U\Delta t),\\ Exit & ∼ & poisson(\mu S\Delta t). \end{array}$$

The simulation process can be described as follows.

$$\begin{array}{ccc} & & S\left(t+\Delta t\right)\leftarrow S\left(t\right)-\sum_{i\in A}NI_{i}+Recruit-Exit,\\ & & I\left(t+\Delta t\right)\leftarrow I\left(t\right)+\sum_{i\in A}NI_{i}-\sum_{i\in A}sgn\left(t+\Delta t-t_{i}^{0}-T_{m}\left(i\right)\right), \end{array}$$ (7)

where *A* is the set of people living with HIV at time *t*, and

$$\begin{array}{c} sgn\left(x\right)=\{\begin{array}{c} 1,\text{if}\;x\geq 0\\ 0,\text{if}\;x<0. \end{array} \end{array}$$ (8)

Suppose there are $\hat{N}$ individuals already infected with HIV when the simulation is terminated. For these $\hat{N}$ individuals, we record critical information for each individual such as by whom he was infected, when he got infected, and at which infection age an infected individual infected others.

### Parameters

In this study we employ some parameter values from literature and others are chosen as follows. In particular, we consider a MSM population of gay men between age 15 and 64. Since the natural death rate is 1/70 [30], we have *μ* = 1/(49*365) + 1/(70*365). Assume that the population has reached a steady state before HIV is introduced, the constant recruitment rate for susceptibles is then *U* = *μ* × *S*(0). A study, in which MSMs who have not been infected with HIV was recruited and then followed by a year, was implemented in Mianyang city of China [31]. From this study we we can get that the mean number of acts of insertive anal intercourse per week is 1.77 for MSM population. Since most individuals infected with HIV had not been diagnosed in their primary stage, we assume that the frequency of high-risk behavior in the primary stage is also 1.77 per week. The research conducted by He et al. [32] showed that the frequency of high-risk behavior in the asymptomatic stage was not affected by the CD4 level and was with a mean of 0.9643 acts per week. During the AIDS stage, the high-risk behaviors reduced significantly, so we will start by assuming that the frequency of high-risk behaviors during the AIDS stage reduced by half compared to the asymptomatic stage, and we will then conduct a sensitive analysis to see how our simulations are affected by this assumption.

We infer the mean condom use rate as 40% from a series of studies [31–34], which leads to *p* = 0.4. The condom efficacy is 0.9 as used by Lou et al in [35]. For the transmission probability, we choose *β*_0_ = 0.01 and *v*_0_ = 10^4.5^ according to the value of *β*_0_ for MSM group estimated by Wilson et al. [4], The definitions and used values of all parameters involved in our simulations are summarized in Table 1.

**Table 1:** Parameters and initial data for between host model.

Parameters	Definition	Value	Source
*S*(0)	Initial value of susceptible population	100000	see text
*μ*	Exit rate	1/(49 × 365) + 1/(70 × 365) day^−1^	see text
*U*	Constant recruitment rate	*μS*(0) day^−1^	see text
*β*_0_	The probability of HIV transmission from a person with baseline viral load *V*_0_	1e-2	[4]
*V*_0_	The baseline viral load	10^4.5^ copies/*ml*	[4]
*c*_1_	Frequency of high risk behavior, Primary stage	1.77/7 day^−1^	[31]
*c*_2_	Frequency of high risk behavior, Asymptotical stage	0.9643/7 day^−1^	[32]
*c*_3_	Frequency of high risk behavior, AIDS stage	0.9643/14 day^−1^	see text
*p*	Intervention measure use rate	0.4	[31–34]
*η*^*c*^	Condom efficacy	0.9	[35]

We use the reference [10] for the maximum survival time *T*_*m*_ obeying Weibull distribution with mean 11(*std = 0.5*) years. In [36], Ghys et. al pointed out that the average net survival in most low and middle income countries has been changed from 9 years to 11 years. A six-year cohort study was implemented in rural areas of Henan by Li et al [37], in which the occurrence time of resistance for 75 individuals with treatment are recorded. Using these data, we estimate the the drug resistance rate using the Least Square Method. It turns out that *r*_*t*_ follows exponential distribution with mean value of 2.23. Infected individuals with higher baseline CD4 level of *B*_*CD*4_ may have a poorer adherence because of the side effect and other reasons [38]. There are lots of studies demonstrated that poor adherence may speed up the emergence of drug resistance [39–42]. Thus, we assume that the earlier treatment is initiated the quicker the drug resistance emerges. We further assume that *r*_*t*_ follows a exponential distribution with mean value 2 if treatment is initiated early. Following the study conducted by Tang et al. [43], we get the value of parameters *s*, *d*, *δ*, *c*. Parameters related to *k*(*t*) and *λ*(*t*) for the within-host model vary individually, and all values or ranges of parameters of the within-host model are described in ***Table A in*** S2 File.

## Results

**Fig 1:**
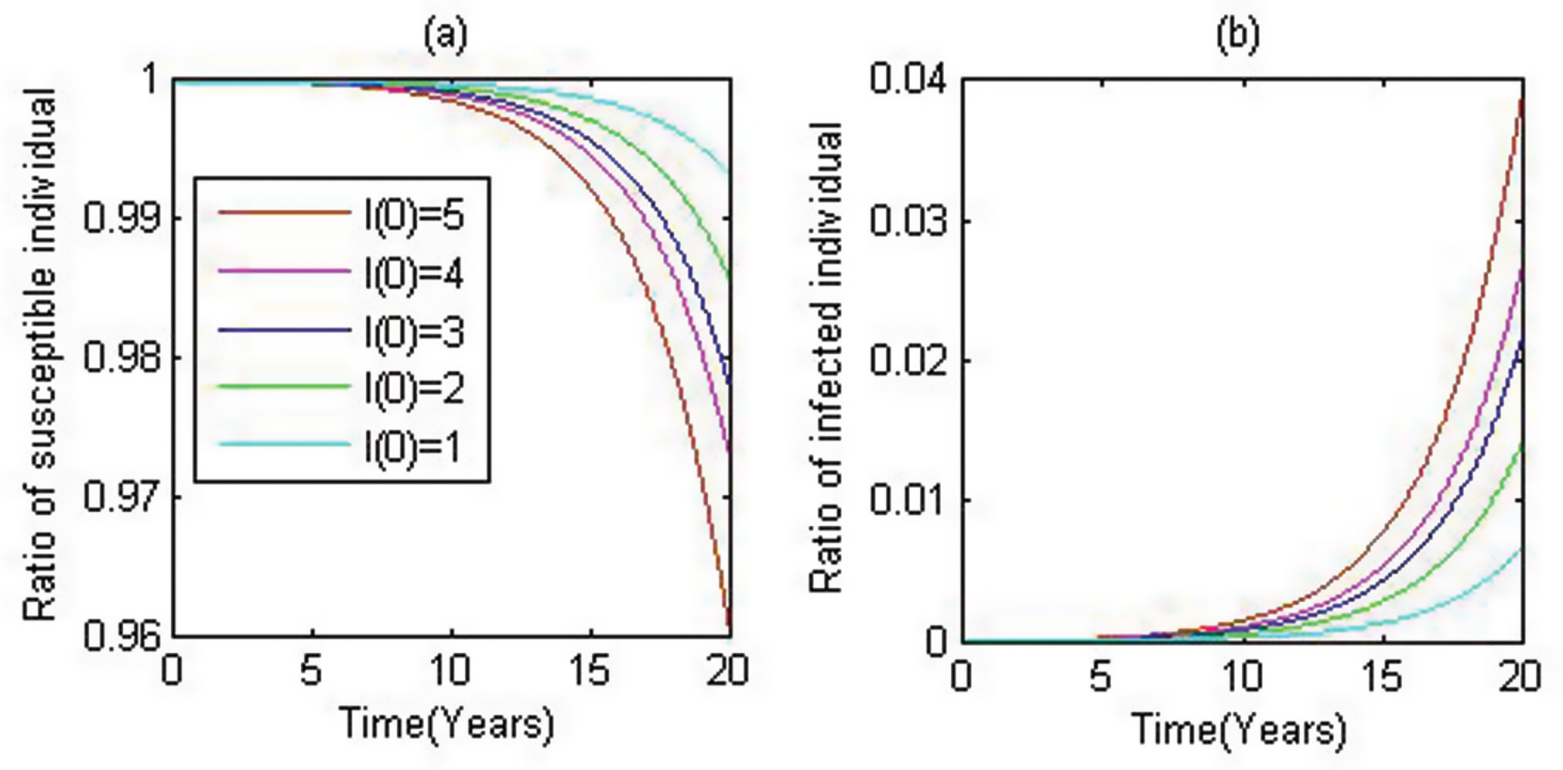
Time series of susceptible and infected individuals. Left panel for the ratio of susceptibles and right panel for the infected individuals, while red, violet, blue, green and cyan represent the cases when 5, 4, 3, 2 and 1 infected individual are initiated.

We initially introduce a single HIV-infected individual into the population with *S*_0_ susceptibles (here we use *S*_0_ = 100,000), and we carry out stochastic simulations based on the individual based model. In the simulations we reported in Fig 1, 50 runs are carried out with the terminal condition of *t* = 20 years. The simulations show the graduate increase of the ratio of HIV-infected individuals, and repeating this procedure with more initial infected individuals gives similar patterns but with greater proportions of infected individuals in the given population.

### Distribution of infection ages

Several studies have indicated that contribution to HIV transmission at the primary stage is relatively high [1, 44, 45]. However, there are also studies suggesting that the contribution to HIV transmission at the primary stage may be overestimated [46–48]. Furthermore, HIV-positive individuals under HAART may live longer due to alleviated illness [49–53], and hence it is important to see how HARRT affects the contribution to HIV transmission at different stages to inform treatment and practical infection control strategies.

**Fig 2:**
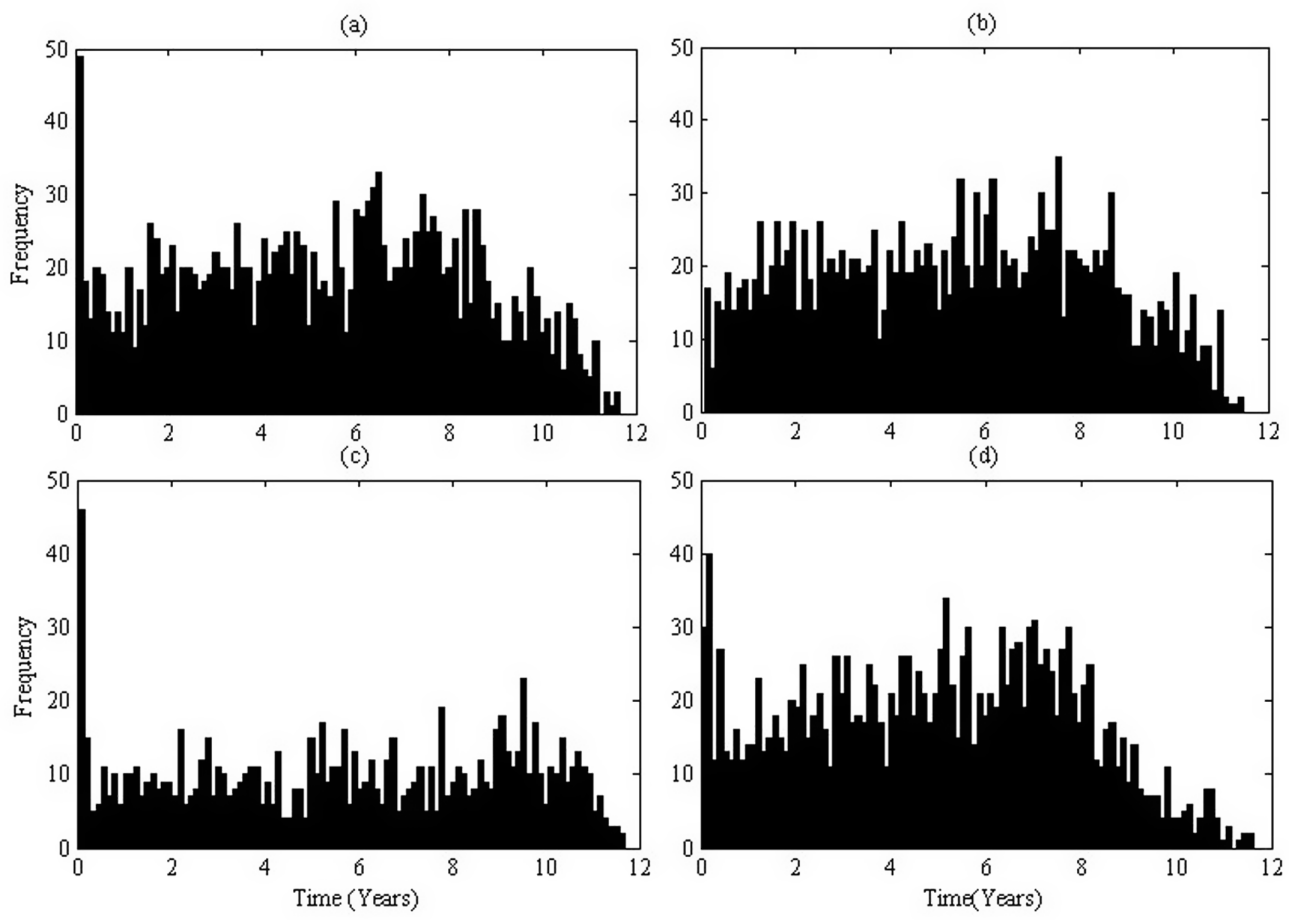
Histogram of the infection age when one infects others. The transmission coefficient is *β*_0_ = 1*e*^−2^. (a). Baseline parameter values listed in **Table A in** S2 File; (b). The infectivity decreased by 1/2 at the primary stage; (c). The infectivity decreased by 1/2 at the asymptomatic stage; (d). The infectivity decreased by 1/2 at the AIDS stage.

In our results reported here, the simulation is suspended once *M* infected individuals are dead. The IBM and stochastic simulations allow us to capture such important information as: who affects whom, and when. Let *I*_*i*_(*D*_*i*_, *a*_*ij*_) be the number of HIV-positive individual infected by the *i*th HIV-positive *D*_*i*_ at his infection age *a*_*ij*_ ∈ [0, *T*_*m*_], where *T*_*m*_ is the maximum survival time). *and its value is 1*. Then

$$\begin{array}{c} \sum_{a_{ij}\in \left[0,T_{m}\right]}I_{i}\left(D_{i},a_{ij}\right)≐{\bar{N}}_{i} \end{array}$$

represents the total number of HIV-positive individuals infected by the *i*th case (*D*_*i*_) during his life span. The histogram of the probability distribution of infection age when one infects the others is assembled in Fig 2 (based on 50 runs with the stated terminal condition (*TC*_*m*_) when *M* = 10. The frequency at the primary stage is the highest, this frequency drops suddenly at the asymptomatic stage and grows gradually with time, and then drops again at the AIDS stage. However, the number of individuals infected by infected individuals at the asymptomatic stage is the greatest due to the long duration of this stage. More precisely, 3.66% of individuals are infected at the primary stage and 81.99% of individuals are infected at the asymptomatic stage. Repeating the simulation with half infectivity at the primary, asymptomatic or AIDS stage gives the subplots Fig 2b, 2c and 2d respectively where the proportions of individuals infected are 1.15%, 85.10%, 13.75% for Fig 2b; 5.67%, 71.00%, 23.30% for Fig 2c; and 4.27%, 88.53%, 7.19% for Fig 2d, respectively.

### Actual reproduction number

The reproduction number provides information on the potential for growth or decline of an epidemic. We here examine the actual reproduction number *R*_*a*_ [54] for HIV transmission that has taken place. This number can be estimated as the average number of secondary cases per infected individual to which the infection was actually transmitted in a population during the infectious period. The estimation of *R*_*a*_ can thus be given as

$$\begin{array}{c} R_{a}=\frac{\sum_{i=1}^{M}\sum_{a_{ij}}I_{i}\left(D_{i},a_{ij}\right)}{M}=\frac{\sum_{i=1}^{M}{\bar{N}}_{i}}{M}. \end{array}$$

**Fig 3:**
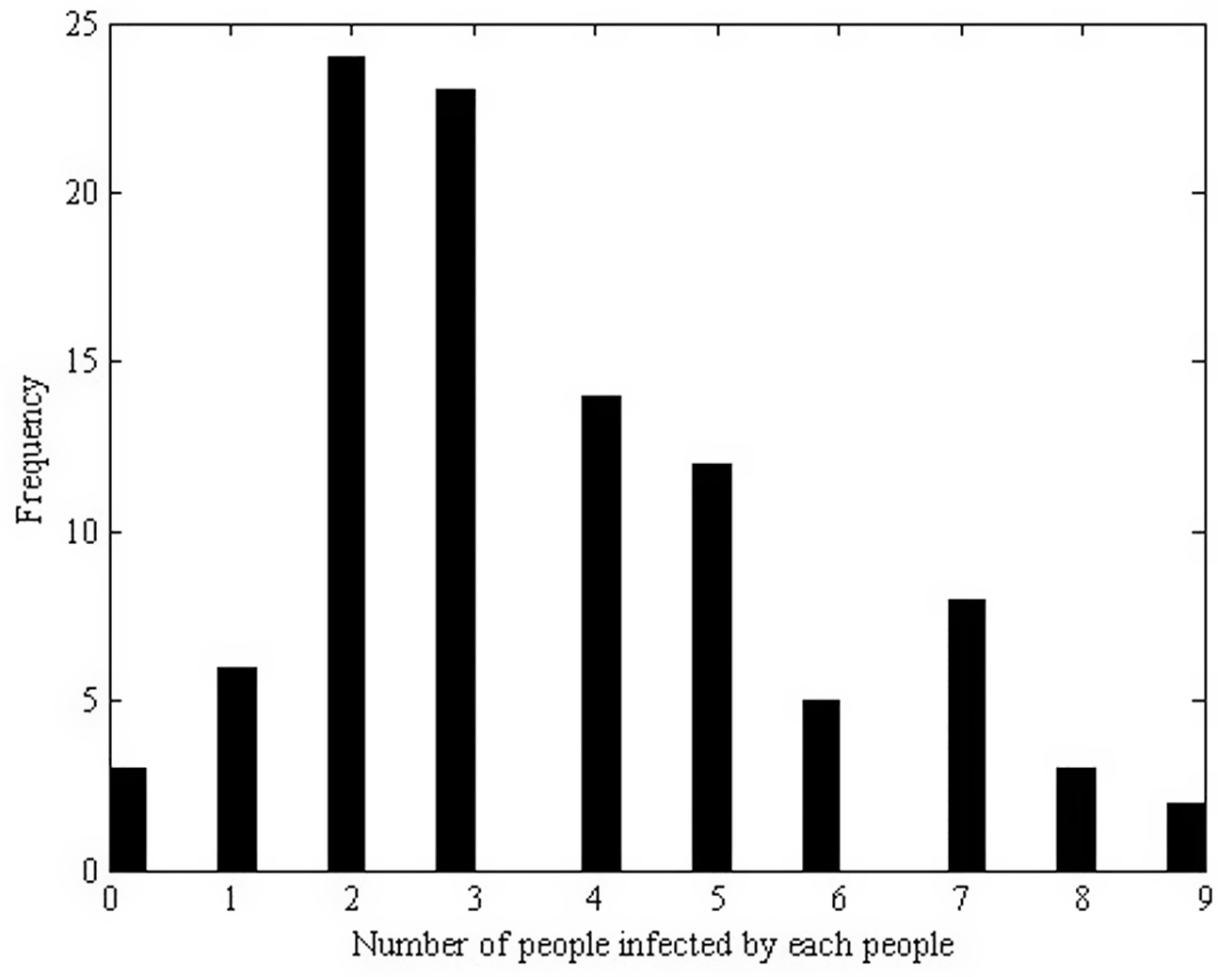
Histogram of the number of secondary cases induced by a single infected individual in a simulation. A simulation with terminal condition of *M* = 100. An infected individual can induce 3.67 secondary cases on average with at most 9 secondary cases during the entire life span.

Here we choose *M* = 100, and the histogram of the number of secondary cases induced by a single infected individual is described in Fig 3 From this we conclude that an infected individual can induce 3.67 secondary cases on average with at most 9 secondary cases during the entire life span. On a close examination, we see that the number of secondary cases induced by an infected individual depends on the survival time of the infected individual, the frequency of high risk behaviors, the infectivity and the level of interventions involved.

In order to reduce the random error and obtain the mean value of the actual reproduction number *R*_*a*_, 50 simulations with *M* = 10 for each simulation are carried out. We also estimate the mean value of *R*_*a*_ to be 3.6320 with standard deviation of 1.92 (95% confidence interval (CI): [3.46, 3.80]). Varying the population size, we can investigate whether the estimated actual reproduction number is sensitive to the population size, and our simulations give the actual reproduction number to be 3.56 (95% CI: [3.40, 3.72]) for *S*_0_ = 10,000, 3.66 (95% CI: [3.49, 3.83]) for *S*_0_ = 50,000 and 3.69 (95% CI: [3.51, 3.87]) *S*_0_ = 200,000. In conclusion, the actual reproduction number is not sensitive to the population size. Hence, throughout the paper we fix *S*_0_ = 100,000.

There are some evidences showing that progression to AIDS is faster among MSMs than among heterosexuals or IDUs [55]. To verify the variations in *R*_*a*_ with the between-host transmission coefficient and progression to AIDS stage in vivo, we vary the transmission coefficients by multiplying a factor *ϵ*, which denote the product of changes of the transmission probability per high risk behavior *β*_0_, the frequency of high-risk behavior *c* and the intensity of interventions *p*(Thus, incidence rate changes to $\epsilon \beta \left(v_{i}\left(t-t_{i}^{0}\right)\right)c\left(t-t_{i}^{0}\right)\left(1-\eta^{c}p\right)S\left(t\right)/N\left(t\right)$). Meanwhile, the rate of progression (survival time *T*_*m*_) is also varied in stochastic simulations. The mean values of actual reproduction number are listed in Table 2. As anticipated, we observe that the longer survival time is, or the greater transmission coefficients are, the greater actual reproduction number *R*_*a*_ is. Note that an infected individual with a short survival time may reproduce more secondary cases, depending on the frequency of high-risk behaviors and the intensity of implemented interventions. Moreover, interventions implemented at different stages may result in difference in efficacy. In particular, we observe that decreasing infectivity at the primary stage (or the AIDS state) by half leads to the mean value of actual reproduction number to be 3.61 (95% CI: [3.45, 3.77])(3.39 (95% CI: [3.22,3.55])). However, if the infection rate is reduced by half at the asymptomatic stage, the actual reproduction number can be reduced to 2.09 (95% CI: [1.96, 2.23]). Therefore, we report that infections at the asymptomatic stage contribute most to the total secondary infections since this stage takes up almost 90% of the survival time.

**Table 2:** Actual Reproduction numbers *R*_*a*_(95% CI).

Mean *T*_*m*_ \ *ϵ*	0.37	0.6	1	1.5
7 years	0.9315([0.82, 1.04])	1.4524([1.34, 1.56])	2.3590([2.23, 2.49])	3.7640([3.59, 3.94])
11 years	1.3870([1.28, 1.50])	2.1766 ([2.04, 2.31])	3.6320([3.46, 3.80])	5.3940 ([5.19, 5.60])

*ϵ* denote the product of changes of the transmission probability per high risk behavior *β*_0_, the frequency of high-risk behavior *c* and the intensity of interventions *p*. Thus, incidence rate changes to $\epsilon \beta \left(v_{i}\left(t-t_{i}^{0}\right)\right)c\left(t-t_{i}^{0}\right)\left(1-\eta^{c}p\right)S\left(t\right)/N\left(t\right)$).

### Effect of HAART on HIV infection

We now consider the effect of different HAART initiation timings and drug efficacy on the actual reproduction number, prevalence, incidence and mortality. In our simulations, HAART may begin when CD4+ T cell counts drop to 500 copies/*μl* (denoted by *B*_*CD*4_ = 500) or 350 copies/*μl* (i.e. *B*_*CD*4_ = 350). Note that infected individuals with higher CD4+ T cell counts may have poorer adherence for a number of reasons including side effects [38]. There are lots of studies demonstrating that poor adherence may speed up the emergence of drug resistant virus variants [39–42]. Therefore, individuals with higher CD4+ T cell counts, if treated early, may develop drug resistance more quickly. For the same reason, individuals with early treatment may progress to AIDS stage quicker after the emergence of drug resistance. On the other hand, individuals with higher CD4+ T cell counts with early HAART may have a better health status and hence better drug sensitivity compared with late HAART. All of these considerations (drug resistance emergence rate, progression rate to AIDS, drug sensitivity) can be incorporated in our within-host model with relevant parameters, and the simulations with these considerations are shown in Figure E-G in S1 File.

Note that in mainland China only first and second line drugs are available and individualized treatment regime has not been implemented. Therefore, an individual patient may still be under the first-line drugs (but with different combinations) even if drug-resistant virus variants emerge. Consequently drug efficacy may either keep at a high level or decline after the emergence of drug resistant variants. We consider these two situations in the following, where relevant parameters are described in ***Table B in*** S2 File.

#### Situation 1. Drug efficacy keeps at a high level after emergence of drug resistant virus variants

We first investigate the effect of various HAART initiation times, represented by the baseline CD4+ T cell counts *B*_*CD*4_, on the actual reproduction number. We focus on two cases: *B*_*CD*4_ = 500 (representing relatively early treatment) and *B*_*CD*4_ = 350 (representing late treatment). It is assumed that individuals with early treatment have better drug sensitivity but progress to AIDS stage at a much quicker speed. Drug resistance is supposed to turn up at a mean of 1/*b* years after treatment is initiated and follow exponential distribution, that is *r*_*t*_ ∼ *Exp*(*b*). 200 runs with terminal condition (*TC*_*m*_, *M* = 1) are carried out and the following four combinations are considered: early/late HAART initiation with quick/slow emergence of drug resistant virus variants. The estimated actual reproduction numbers are listed in Table 3 Situation 1. It shows that in the case where *B*_*CD*4_ = 500 the estimated actual reproduction numbers are consistently less than those without treatment. While in the case where *B*_*CD*4_ = 350 the values of actual reproduction numbers are greater than those without treatment. Therefore, early treatment indeed leads to less *R*_*a*_ and hence less new infections compared to later treatment no matter when the drug resistant variants emerge.

**Table 3:** Actual Reproduction numbers for Situation 1 and Situation 2.

*B*_*CD*4_	Situation 1		Situation 2	
	*r*_*t*_	*R*_*a*_	*r*_*t*_	*R*_*a*_
500	*Exp*(2)	2.98	*Exp*(2)	4.00
500	*Exp*(0.4482)	2.30	*Exp*(0.4482)	3.83
350	*Exp*(2)	3.63	*Exp*(2)	3.81
350	*Exp*(0.4482)	3.63	*Exp*(0.4482)	3.47

Parameter values for situation1 and situation 2 are chosen from ***Table B in*** S2 File Situation 1 and Situation 2, respectively.

**Fig 4:**
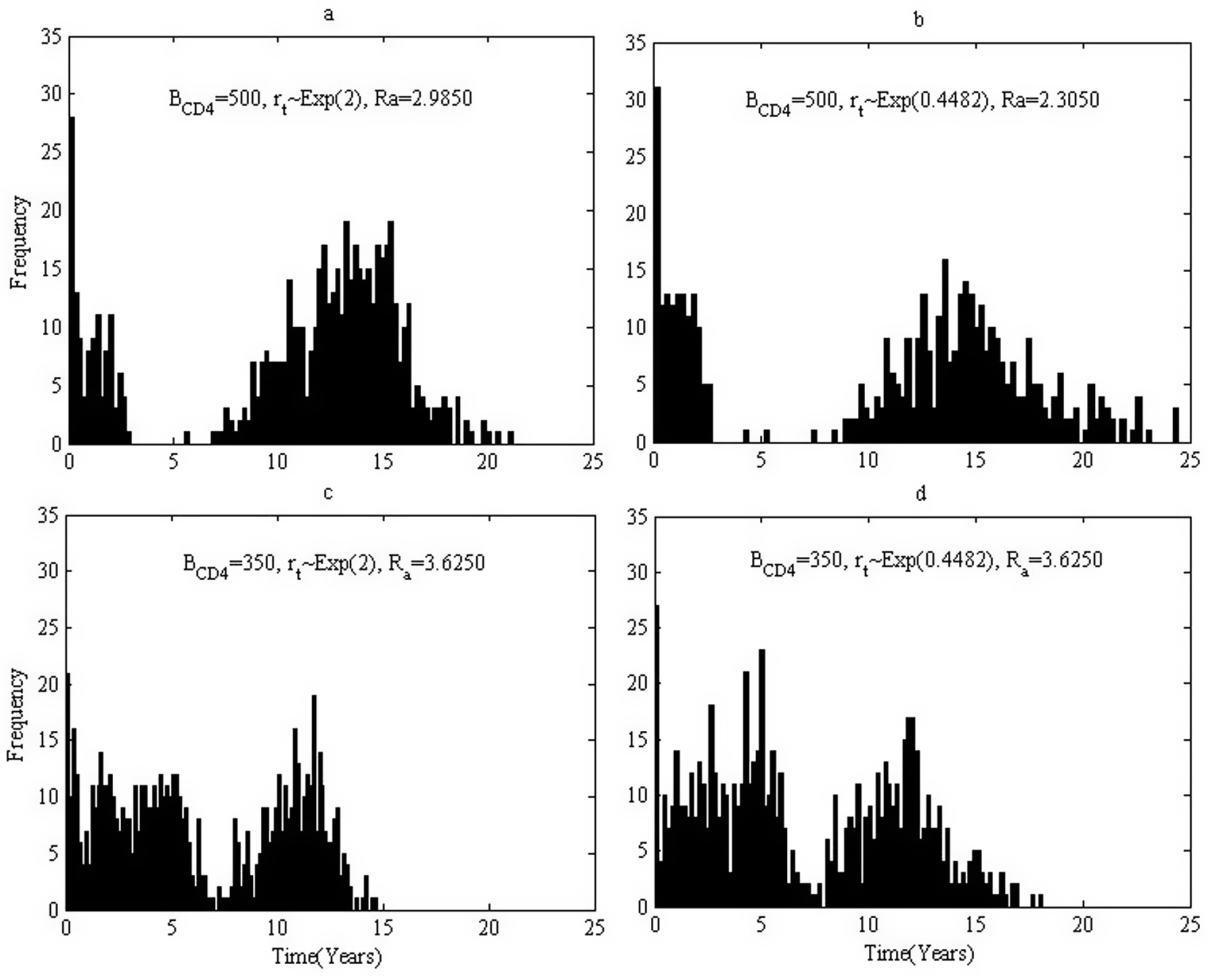
Histogram of the infection age when one infects others for various baseline CD4 level. Parameters are chosen from **Table B in** S2 File situation 1. (a). Treatment started when CD4 cell count is less than 500, *r*_*t*_ follows the exponential distribution with mean 0.5, the basic reproduction number is 2.9850. (b). Treatment started when CD4 cell count is less than 500, *r*_*t*_ follows the exponential distribution with mean 2.23, the basic reproduction number is 2.3050. (c). Treatment started when CD4 cell count is less than 350, *r*_*t*_ follows the exponential distribution with mean 0.5, the basic reproduction number is 3.6250. (d). Treatment started when CD4 cell count is less than 350, *r*_*t*_ follows the exponential distribution with mean 2.23, the basic reproduction number is 3.6250.

The infection ages when those 200 infected individuals(200 simulations with each simulation 1 individual) induce new infections in multiple simulations are recorded and the distribution of infection ages are shown in Fig 4. For the case where *B*_*CD*4_ = 500, infected individuals can keep at a relatively low level of infectivity for about 5 years due to effective HAART, and hence the tracked infected individuals barely induce secondary cases (Fig 4a and 4b). However, the frequency displays a peak during 10 to 20 years after infection. This is because viral loads rebound due to emergence of drug resistant variants. Note that few individuals can survive more than 25 years as we assume only first-line drugs are available. It follows from Fig 4c and 4d for the case where *B*_*CD*4_ = 350 HAART can only effectively suppress viral loads for about one year. Comparing Fig 4a with 4b implies that quick emergence of drug resistant variants leads to higher actual reproduction number when *B*_*CD*4_ = 500(early treatment), while Fig 4c and 4d implies the timing of emergence of drug resistant variants has little impact on the new infections when *B*_*CD*4_ = 350(late treatment). Moreover, it follows from Fig 4a and 4c (Fig 4b and 4d) that early treatment induces low actual reproduction number and hence less new infections no matter how quick the drug resistant variants emerge.

**Fig 5:**
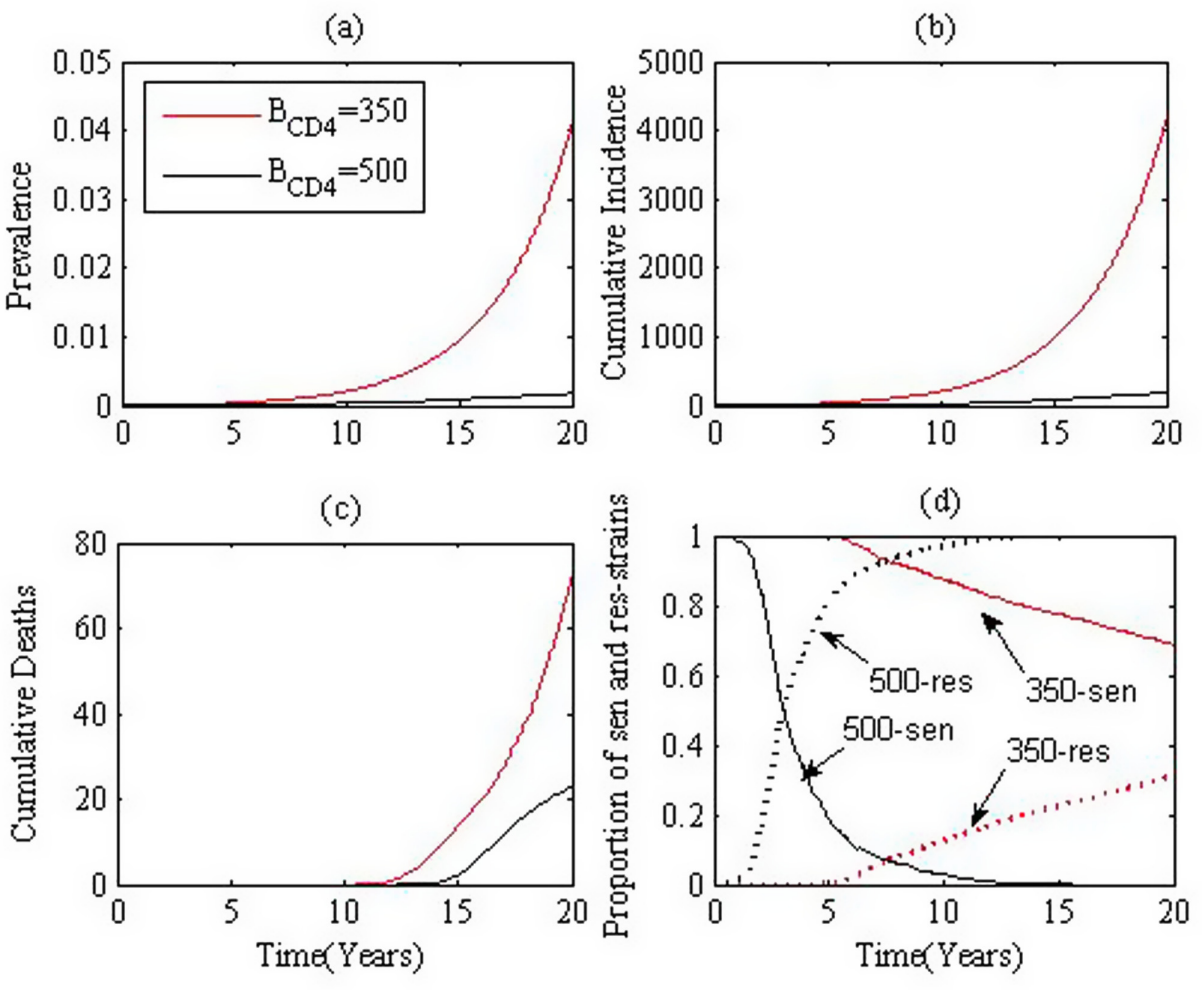
Effect of HAART initiation times. (a). prevalence, (b). incidence, (c). cumulative deaths and (d). the proportion of resistant strain. Black and red curves denote treatment initiated when CD4+ T cell counts drop to 500 and 350, respectively. Parameters are chosen from **Table B in** S2 File situation 1.

We now consider the effect of HAART on transmission at the population level. We start with the case where we assume that individuals with early treatment have better drug sensitivity but progress to AIDS stage at a much quicker speed, and the drug resistance turns up at a much quicker rate. We introduce 10 infected individual to a fully susceptible population (*S*_0_), and then perform simulations for 20 years. 50 runs are carried out and the mean prevalence, cumulative incidence, cumulative deaths and ratio of individuals with resistant virus are shown in Fig 5a, 5b, 5c and 5d respectively. The black and red curves denote the simulation results for *B*_*CD*4_ = 500 and *B*_*CD*4_ = 350, respectively. Here, we assume that drug resistance turns up at a mean of 1/2 and 1/0.4482 years after treatment is initiated for *B*_*CD*4_ = 500 and *B*_*CD*4_ = 350, respectively. Fig 5 indicates that early treatment leads to a lower prevalence, less new infections (incidence) and fewer deaths. It is worthy noting that the proportion of infected individuals with drug resistant virus increases more rapidly under early treatment than late treatment.

We mention that in the case when individuals with early or late treatment have the same sensitivity and progress to AIDS stage at the same speed, we observe similar results(not shown here): early treatment leads to less *R*_*a*_, lower prevalence, less new infections (incidence) and fewer deaths compared to later treatment.

#### Situation 2: Drug efficacy drops substantially after emergence of drug resistant virus variants

**Fig 6:**
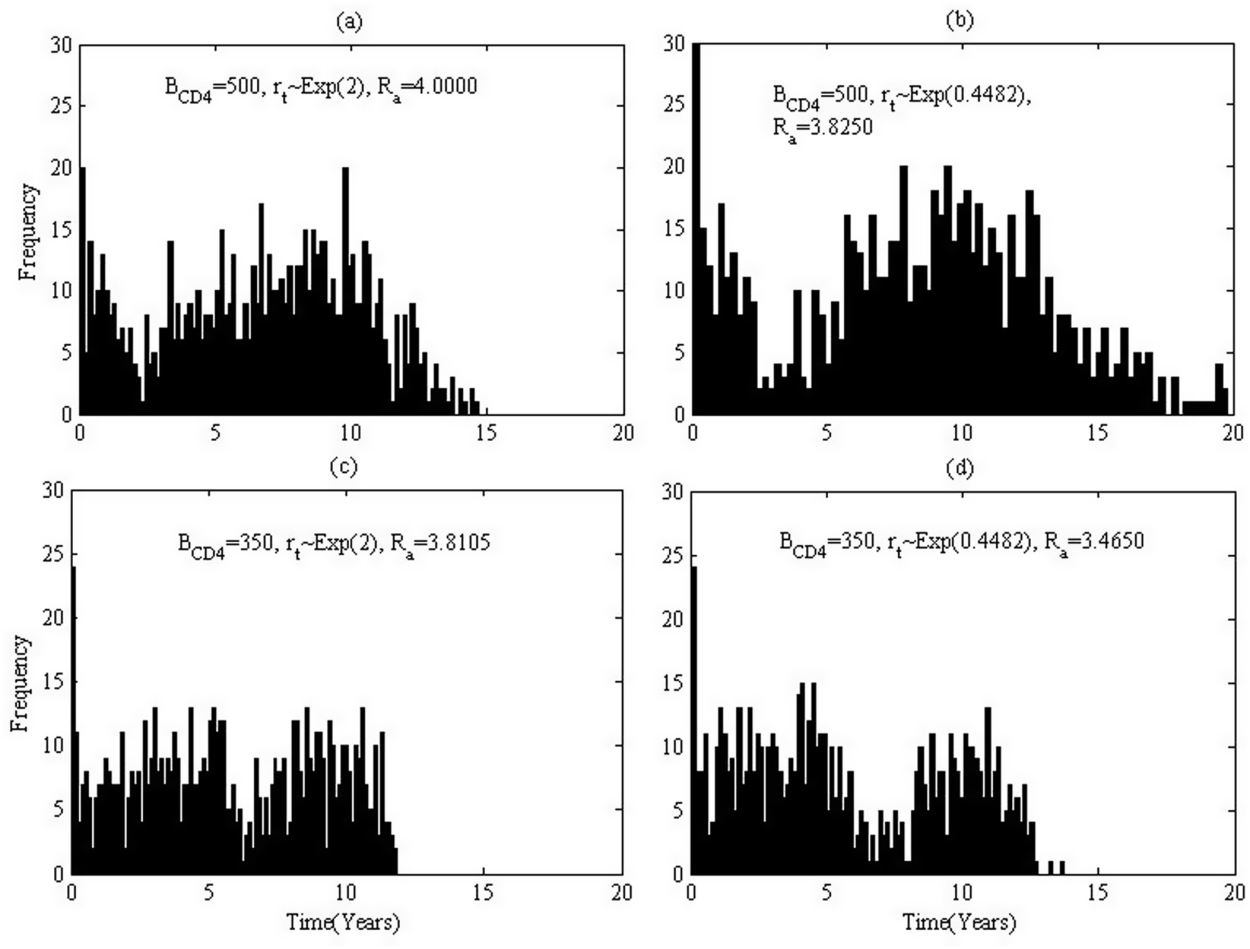
Histogram of the infection age when one infects others for various baseline CD4 level. Parameter values are chosen from **Table B in** S2 File situation 2. (a). Treatment started when CD4 cell count is less than 500, *r*_*t*_ follows the exponential distribution with mean 0.5, the basic reproduction number is 4.0000. (b). Treatment started when CD4 cell count is less than 500, *r*_*t*_ follows the exponential distribution with mean 2.23, the basic reproduction number is 3.8250. (c). Treatment started when CD4 cell count is less than 350, *r*_*t*_ follows the exponential distribution with mean 0.5, the basic reproduction number is 3.8150. (d). Treatment started when CD4 cell count is less than 350, *r*_*t*_ follows the exponential distribution with mean 2.23, the basic reproduction number is 3.4650.

Similarly, basing on the assumption that individuals with early treatment have better drug sensitivity but progress to AIDS stage at a quicker speed, we carry out four sets of simulations for different initiation times and different times of emergence of drug resistant virus variants. The simulated actual reproduction numbers are listed in Table 3 situation 2. It is interesting to observe that the actual reproduction numbers for *B*_*CD*4_ = 500 are greater than those for *B*_*CD*4_ = 350 under a given rate of emergence of drug resistant variants. That is because individuals with early treatment may lead to lower infectivity during earlier and shorter duration, but can result in longer survival, compared with those with late treatment. This conclusion is also validated by comparing Fig 6a and 6c (or Fig 6b and 6d) that early treatment induces greater actual reproduction number and hence more new infections no matter how quickly the drug resistant variants emerge. It is interesting to notice that this conclusion is in contrast to Situation 1.

It is easy to understand that the values of actual reproduction number for situation 2 (Fig 6a–6c) are greater than those for situation 1(Fig 4a–4c), since the drug efficacy drops substantially after emergence of drug resistant virus variants for situation 2. However, it is important to mention that for case *B*_*CD*4_ = 350 with *r*_*t*_ ∼ *Exp*(0.4482) the actual reproduction number of situation 1 (Fig 4d) is larger than that for situation 2 (Fig 6d). Compared with Fig 6d with Fig 4d, we notice that people can survive for a much longer period in situation 1. That is, people in situation 1 can decrease their infectivity much more significantly but live for a much longer time. People in situation 2, in contrast, can decrease their infectivity much lower but live for a much shorter period. Consequently, there is a *trade-off* between lowered infectivity and extended survival time for an individual with treatment.

**Fig 7:**
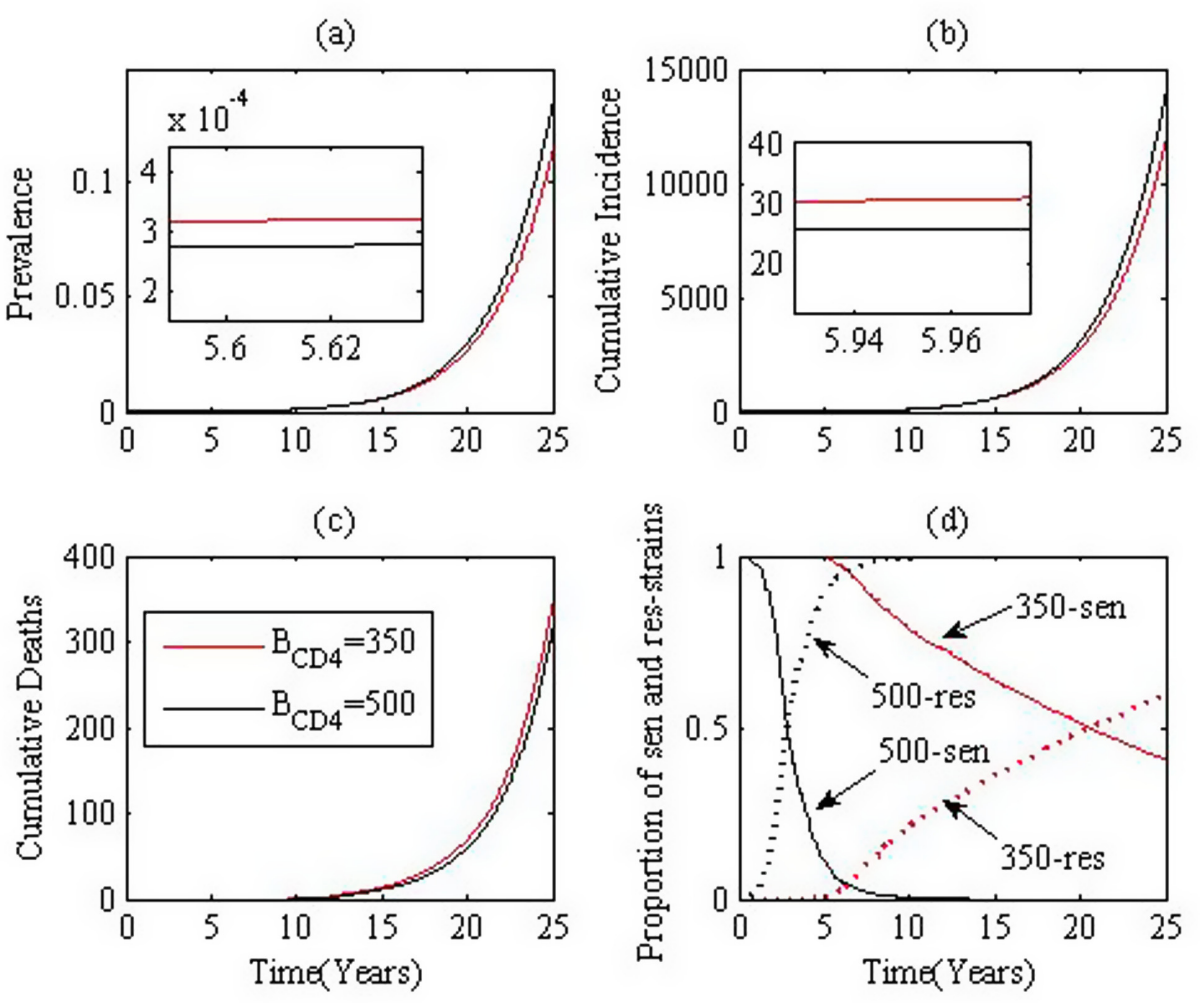
Effect of times of initiation of treatment. (a). prevalence, (b). incidence, (c). cumulative deaths and (d). the proportion of resistant strain. Black and red curves denote treatment initiated when CD4+ T cell counts drop to 500 and 350, respectively. Parameters are chosen from **Table B in** S2 File situation 2.

With 50 runs for 25 years where 5 infected individuals are introduced into a fully susceptible group (*S*_0_), we produce the mean prevalence, cumulative incidence, cumulative deaths and ratio of infected individuals with resistant virus, as shown in Fig 7a, 7b, 7c and 7d respectively. We emphasize that during the first several years after the initiation of treatment, early treatment leads to lower prevalence, less cumulative incidences, while after the transition years, early treatment leads to higher prevalence and greater cumulative incidences. Similarly, the proportion of individuals infected with drug resistant type virus increases more rapidly than late treatment.

#### Remark

If individuals with early or late treatment have the same sensitivities and progress to AIDS stage at the same speed, we can get that early treatment and late treatment lead to very similar actual reproduction number, prevalence and cumulative incidence.

## Discussions and conclusions

HIV epidemiological models of population dynamics focus on the temporal variation of the numbers of susceptible and infected individuals at the population level, whereas viral dynamics models concentrate on the within-host dynamics at the individual level. In this study, we proposed an individual based model that explicitly links the within-host dynamics to between-host transmission in order to examine the comprehensive impact of early HAART on HIV infection at the population level. Our viral dynamics simulation model produces viral dynamics mimicking the observed progression during the entire life span [25, 26, 56–58]. There are some models formulated at both within- and between-host levels [17–23]. Coombs and Gilchrist [17, 18] evaluated the direction of natural selection (in the study of evolution of virulence) by using a nested model. Feng et al. [23] linked the epidemiological and immunological dynamics through an environmental component, and hence their model is more appropriate for an environmentally-driven infectious disease. However, our simulation models are appropriate to many infectious diseases and the individual base simulations allow us to track any HIV infected individual and record detailed information on *who infected whom and when*.

Using stochastic simulations, we investigated contributions at different stages -primary, asymptomatic, and AIDS stage- to HIV new infections. Simulation results showed that 3.55% of individuals are infected at the primary stage, which is much lower than the estimation obtained by Brenner et al. and Wawer et al. [1, 45, 46]. Note that here the primary stage is determined by the within-host model, i.e. between initial infection and the first time when the viral load decreases to the lowest value. The mean duration of the primary stage is around 3 months, which agrees with that determined by Zhou et al. [28]. However, Brenner et al. [45] and Wawer et al. [1] defined the early stage as less than 6 months after seroconversion, which is much longer than we obtained here. That is why less contribution at primary stage was obtained in our simulations. When we introduced 5 infected individuals in a fully susceptible population (*S*_0_ = 100,000), about 4% of the total population is HIV-positive 20 years later. We note that the first AIDS patient among MSM was diagnosed in 1989 in mainland China (several HIV-positive individuals had already been there with only one diagnosed) and the prevalence of infection in MSM was 3.4% in 2009 based on the surveillance data [59].

The basic reproduction number *R*_0_, defined as the average number of secondary cases induced by a typical infectious individual during its average infectious period in a wholly susceptible [60], is the most widely used epidemiological measurement of the transmission potential in a situation where all persons are susceptible. In contrast, the actual reproduction number for an epidemic that has occurred, is defined as the average number of secondary cases per infected individual to which the infection is actually transmitted during the infectious period in a population. This was proposed for HIV/AIDS [54]. In the actual spread of HIV, it has been practically impossible to establish actual chains of who infected whom. However, our individual based simulation model provide information including not only the number of persons to whom one infected individual spreads the disease, but also at what age the infection takes place. Therefore, we could estimate the mean actual reproduction number for HIV epidemic as 3.63 (95% CI [3.46, 3.80]) for MSM in China. This estimation is in agreement with that (*R*_0_ = 3.9296) for the MSM group using the deterministic differential equations [35, 61]. It is also in agreeable range with those obtained in a published study of the data among homosexual/bisexual men from European countries: France (3.38—3.81), Western Germany (3.43—4.08) and UK (3.38—3.96) [62]. Moreover, this actual reproduction number is sensitive to the transmission coefficient at population level, as the transmission model predicted [63–65], and the mean survival time at the individual level. It needs to be mentioned that we studied the HIV transmission among MSM in mainland China as a whole. We further note that the actual reproduction numbers for MSM groups of other areas may be various but the proposed method in this study can be used to estimate actual reproduction number for MSM group of any other areas provided other parameter values being available.

We examined the effects of time of initiating treatment, time of emergence of drug resistant virus variants and drug efficacy on the actual reproduction number *R*_*a*_, prevalence, cumulative incidences, cumulative deaths and proportion of individuals infected with drug-resistant virus. We considered the case where drug efficacy is at a relatively high level after emergence of drug resistant variants, and we concluded that earlier treatment leads to a less actual reproduction number, lower prevalence, less incidences and fewer deaths. Hence early initiation of treatment is beneficial to controlling HIV new infections, cumulative incidence and hence curbing disease transmission in this case. However, our simulations also show that if drug efficacy after the emergence of drug resistant virus variants is relatively low, then earlier initiation of treatment may lead to greater actual reproduction number, higher prevalence and larger incidences and deaths. In such a scenario early initiating treatment may not result in the HIV new infections decline but may actually lead to higher new infections, prevalence and cumulative incidence. We noticed a similar conclusion that HAART has a limited effect on HIV incidence in MSM [15].

In both situations, we observed that the proportion of individuals infected with drug resistant virus grows very rapidly. This inevitably yields that the third or successive generation cases are with intrinsic resistant variants. Then the HIV infection control would be a huge public health challenge if effective drugs for the resistant strain are not available in time. Therefore, early initiation of HAART as prevention should be adopted with great care, especially in the settings where effective drugs are not available or not sufficient. Whether treatment can keep at a high efficacy level or not is uncertain, thus the decision for initiating treatment is crucial [12, 13]. In particular, in mainland China where only second-line drugs are available, the strategy of treatment as prevention should not be implemented incautiously.

We caution that in reaching the observations, we assumed that high-risk behavior is unchanged after HAART is initiated. The implications of our simulations should also be interpreted with care. Note that in the epidemiological process we chosen parameter values associated with MSMs. We believe our model approach and simulation strategies are applicable to other high-risk population groups. Note also that we did not consider the rate of diagnose or coverage of treatment, which may affect the actual reproduction numbers. We noted that there are some opinions [55] that disease progression in the MSM group may be quicker than other high-risk groups, however we could not find definition information about the survival time for MSMs in mainland China. We were forced to take the mean survival time for the MSMs without treatment as 11 years, which was estimated for other high-risk groups [10]. This assumption of the mean survival time does not affect the distribution of infection ages or the comprehensive effect of treatment but may lead to under or over-estimated actual reproduction number. Despite these caveats, our simulation results suggest early initiation of HAART may lead to higher number of secondary cases, depending on the level of drug efficacy after emergence of drug resistant virus variants, the frequency of high-risk behaviors after initiation of HAART and etc, hence early HAART should be implemented with great caution.

## Supporting Information

S1 File Effect of parameter values on HIV progression in vivo. Figure A, A simulation of typical course of human immunodeficiency virus infection. Figure B, Simulations of HIV disease progressions with different shape, scale and location parameters. Figure C, 1000 simulations of typical course of human immunodeficiency virus infection without ART. Figure D, Duration of different infection stages. Figure E, Effects of key factors on the HIV disease progression. Figure F, Effects of key factors on the HIV disease progression. Figure G, Effects of key factors on the HIV disease progression.
(PDF)

S2 File Parameter values for within-host model. Table A, Parameter values for within-host model. Table B, Parameter values in situation 1&2.
(PDF)
