# Supplementary material for: Early HAART Initiation May Not Reduce Actual Reproduction Number and Prevalence of MSM Infection: Perspectives from Coupled within- and between-Host Modelling Studies of Chinese MSM Populations
Source: PLoS One. 2016 Mar 1;11(3):e0150513. doi: 10.1371/journal.pone.0150513 (PMC4773120; doi:10.1371/journal.pone.0150513)
Supplement: S1 File — Figure A, A simulation of typical course of human immunodeficiency virus infection. Figure B, Simulations of HIV disease progressions with different shape, scale and location parameters. Figure C, 1000 simulations of typical course of human immunodeficiency virus infection without ART. Figure D, Duration of different infection stages. Figure E, Effects of key factors on the HIV disease progression. Figure F, Effects of key factors on the HIV disease progression. Figure G, Effects of key factors on the HIV disease progression. (PDF) [file pone.0150513.s001.pdf]

S1 File. Effect of parameter values on HIV progression in vivo.

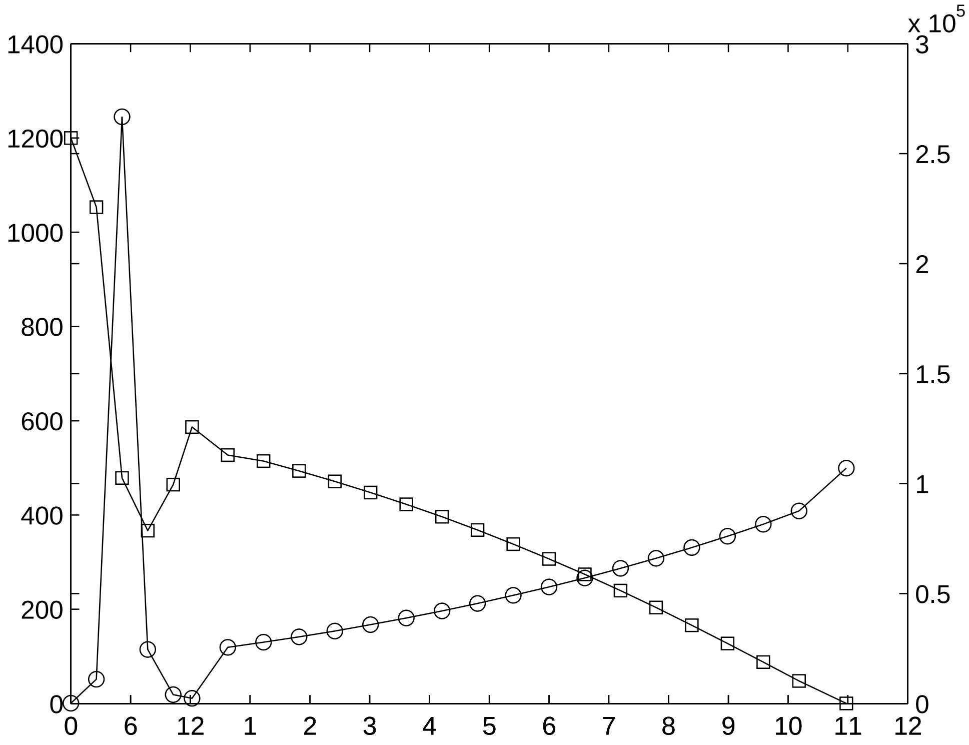

Figure A. A simulation of typical course of human immunodeficiency virus infection: CD4+ T cells($-\Box$) and viral load ($-\circ$). $\beta_k = 3400, \alpha_k = 1.1, \beta_\lambda = 5000, \alpha_\lambda = 0.04, T_m = 11 \times 365$.

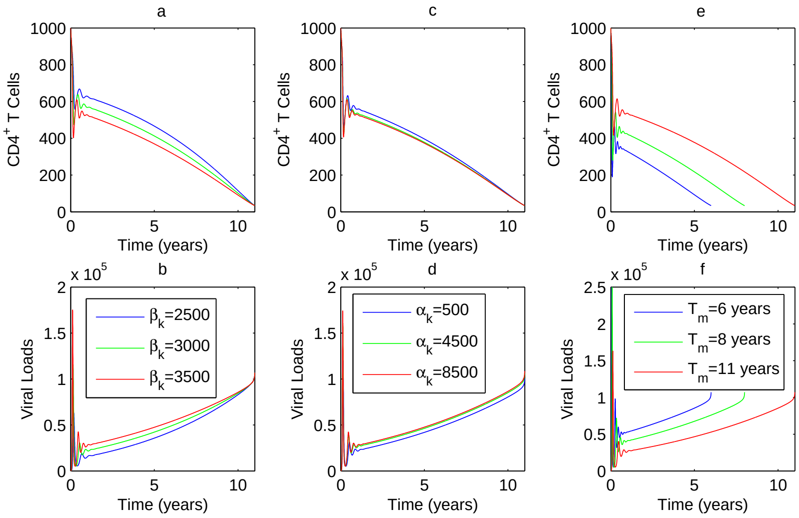

Figure B. Simulations of HIV disease progressions with different shape (α_k), scale (β_k) and location parameters (T_m). $\beta_k = 3400, \alpha_k = 1.1, \beta_\lambda = 5000, \alpha_\lambda = 0.04, T_m = 11 \times 365$.

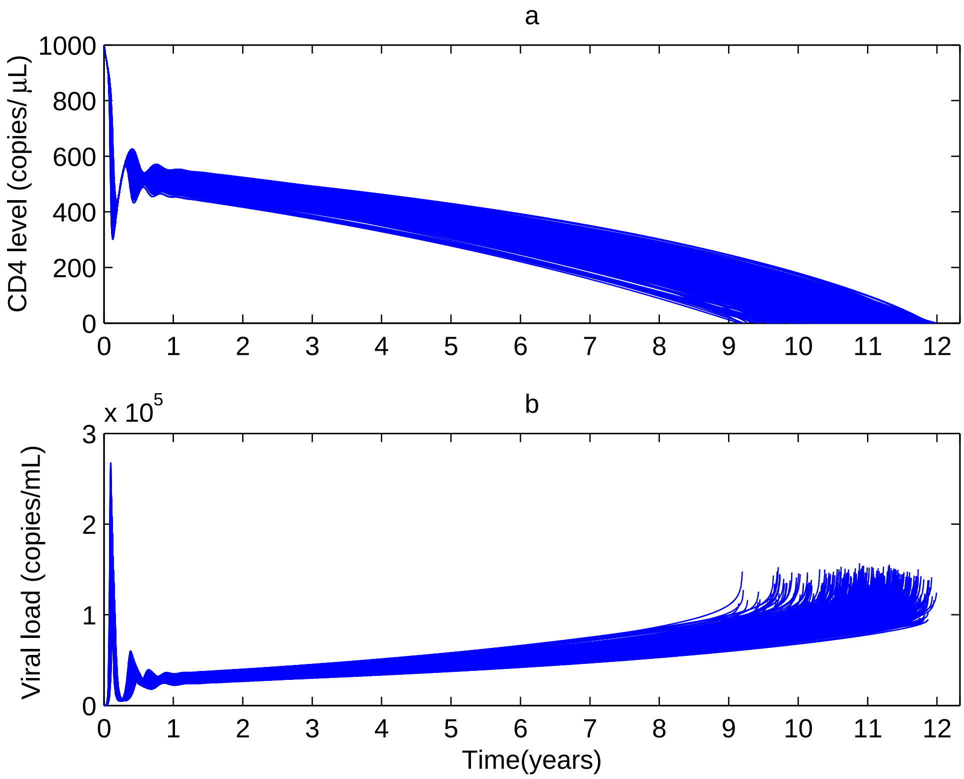

Figure C. 1000 simulations of typical course of human immunodeficiency virus infection without ART.

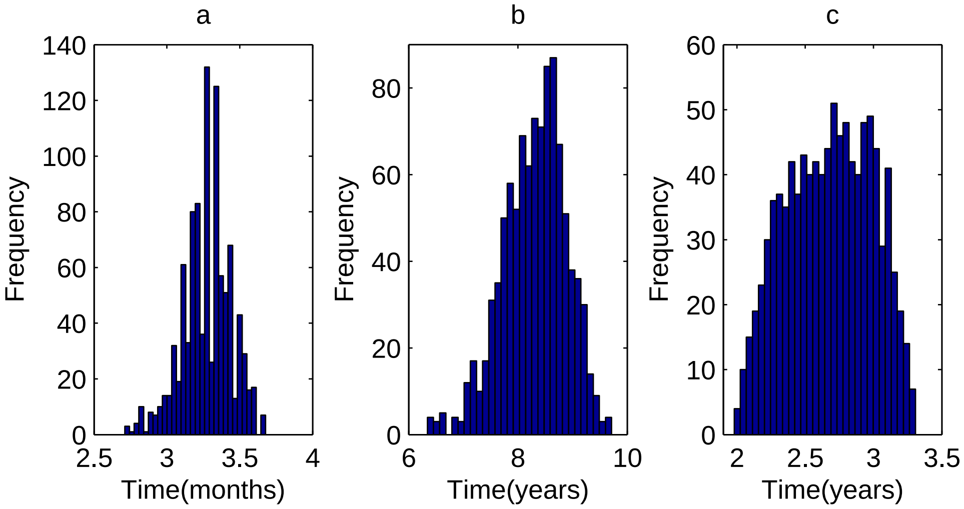

Figure D. Duration of different infection stages. a. duration of primary stage; b. duration of asymptotical stage; c. duration of AIDS stage.

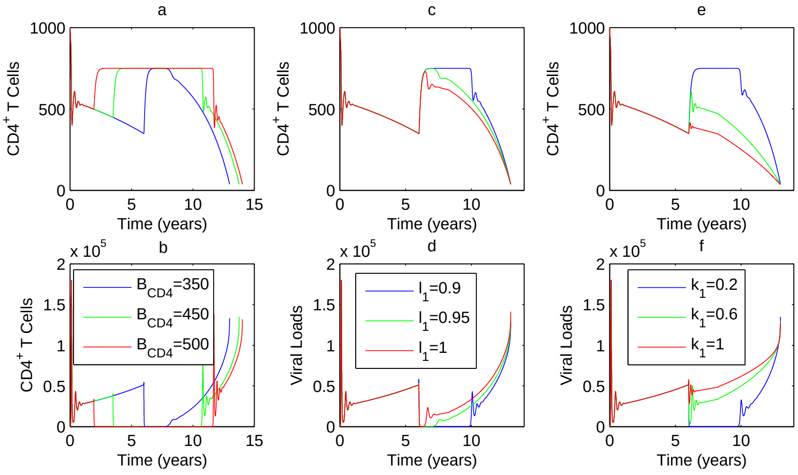

Figure E. Effects of key factors on the HIV disease progression. a-b. Baseline CD4$^+$ cell counts with $B_{CD4} = 350, 450, 500$; c-d. $l_1 = 0.9, 0.95, 1$, $l_1 = l_2$; e-f. $k_1 = 0.2, 0.6, 1$, $k_1 = k_2$. $\beta_k = 3400, \alpha_k = 1.1, \beta_\lambda = 5000, \alpha_\lambda = 0.04$, $T_m = 11$ years, $\tau_m = 35$ years.

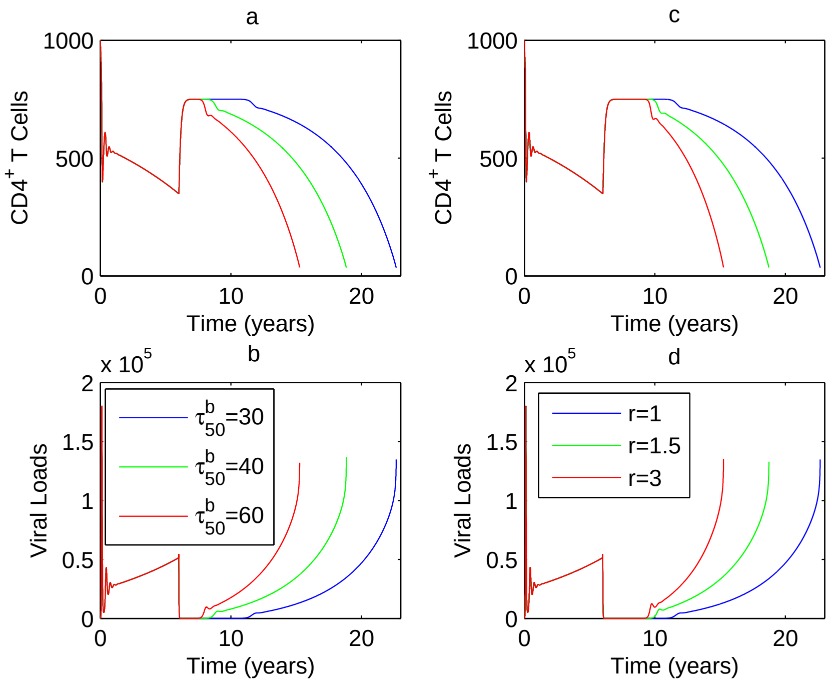

Figure F. Effects of key factors on the HIV disease progression. a-b. $\tau^b_{50} = 30, 40, 60$, $r = 1$; c-d. $r = 1, 1.5, 3$, $\tau^b_{50} = 30$. $\beta_k = 3400$, $\alpha_k = 1.1$, $\beta_\lambda = 5000$, $\alpha_\lambda = 0.04$, $T_m = 11$ years, $\tau_m = 35$ years.

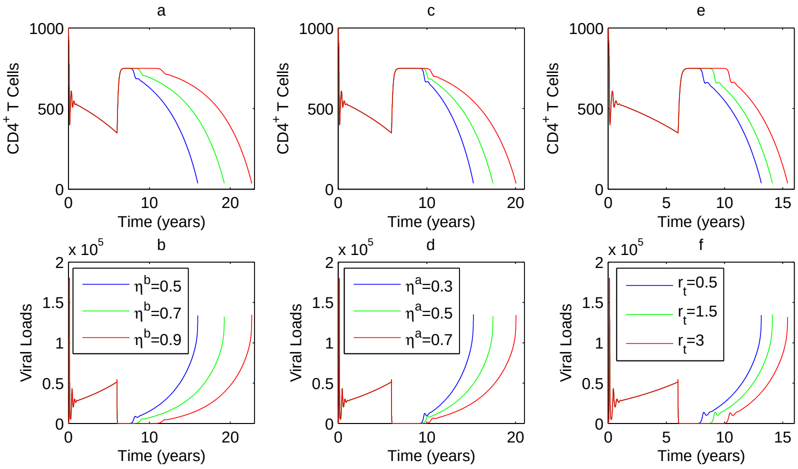

Figure G. Effects of key factors on the HIV disease progression. a-b. $\eta^b = 0.5, 0.7, 0.9$, $\eta^a = \eta^b$; c-d. $\eta^a = 0.3, 0.5, 0.7$, $\eta^b = 0.9$; e-f. $r_t = 0.5, 1.5, 3$. $\beta_k = 3400$, $\alpha_k = 1.1$, $\beta_\lambda = 5000$, $\alpha_\lambda = 0.04$, $T_m = 11$ years, $\tau_m = 35$ years.
