# Supplementary material for: Early HAART Initiation May Not Reduce Actual Reproduction Number and Prevalence of MSM Infection: Perspectives from Coupled within- and between-Host Modelling Studies of Chinese MSM Populations
Source: PLoS One. 2016 Mar 1;11(3):e0150513. doi: 10.1371/journal.pone.0150513 (PMC4773120; doi:10.1371/journal.pone.0150513)
Supplement: S2 File — Table A, Parameter values for within-host model. Table B, Parameter values in situation 1&2. (PDF) [file pone.0150513.s002.pdf]

S2 File. Parameter values for within-host model

Table A. Parameter values for within-host model

Parameter	Definition	Value/Range
s	Rate of supply of CD4+T cell from precursors	15
d	Death rate of uninfected CD4+ T cells	0.02 day^{-1}
δ	Death rate of infected CD4+ cells	0.45 day^{-1}
c	Death or clearance rate of free virus	2.4 day^{-1}
k	Infection rate per virion before treatment	2.1818e-7
k^b	Infection rate per virion after treatment (before emergence of drug resistance)	$l_1 k$
k^a	Infection rate per virion after treatment (after emergence of drug resistance)	$l_2 k$
λ	Number of free virus produced by lysing a CD4+ T cell before treatment	3928.6
λ^b	Number of free virus produced by lysing a CD4+ T cell after treatment (before emergence of drug resistance)	$l_1\lambda$
λ^a	Number of free virus produced by lysing a CD4+ T cell after treatment (after emergence of drug resistance)	$l_2\lambda$
β_k	Scale parameter related to infection of target cells before treatment	U[3300,3500]
β_k^b	Scale parameter related to infection of target cells after treatment (before emergence of drug resistance)	$k_1\beta_k$
β_k^a	Scale parameter related to infection of target cells after treatment (after emergence of drug resistance)	$k_2\beta_k$
β_λ	Scale parameter related to virus production before treatment	U[4000,6000]
β_λ^b	Scale parameter related to virus production after treatment (before emergence of drug resistance)	β_λ
β_λ^a	Scale parameter related to virus production after treatment (after emergence of drug resistance)	β_λ
α_k	Shape parameter related to infection of target cells before treatment	U[0.8,1.1]
α_k^b	Shape parameter related to infection of target cells after treatment (before emergence of drug resistance)	α_k
α_k^a	Shape parameter related to infection of target cells after treatment (after emergence of drug resistance)	α_k
α_λ	Shape parameter related to virus production before treatment	U[0.01,1]
α_λ^b	Shape parameter related to virus production after treatment (before emergence of drug resistance)	α_λ
α_λ^a	Shape parameter related to virus production after treatment (after emergence of drug resistance)	α_λ
T_m	Maximum life span without therapy	W(11.22,27.51)
τ_m	Maximum Life expectancy after infection	U[30,40] years
τ_{50}^b	Drug sensitivity of combination therapy before emergence of drug resistance	Table B
τ_{50}^a	Drug sensitivity of combination therapy after emergence of drug resistance	$r\tau_{50}^b$
η^b	Drug efficacy of combination therapy before emergence of drug resistance	U[0.8,1]
η^a	Drug efficacy of combination therapy after emergence of drug resistance	Table B
B_{CD4}	CD4 level when treatment initiated	500 or 350
T_t	Time to initiate treatment after infection	Determined by B_{CD4}
T_r	Time of the emergence of drug resistance	$T_t + r_t$
r_t	Resistance emergence rate	Exp(2),Exp(0.4482)

The values of parameters s, d, δ, c are determined following the study of Tang et al. [1]. Parameters related to $k(t)$ and $\lambda(t)$ for the within-host model vary individually.

Table B. Parameter values in situation 1 & 2

Parameters	Situation 1		Situation 2	
	$B_{CD4} = 350$	$B_{CD4} = 500$	$B_{CD4} = 350$	$B_{CD4} = 500$
l_1	$U(0.935, 0.940)$	$U(0.935, 0.940)$	$U(0.935, 0.940)$	$U(0.935, 0.940)$
l_2	$U(0.935, 0.940)$	$U(0.935, 0.940)$	$U(1, 1)$	$U(1, 1)$
τ_{50}^b	$U(30, 50)$	$U(20, 30)$	$U(50, 60)$	$U(20, 30)$
r	$U(2, 3)$	$U(2, 3)$	$U(4, 5)$	$U(4, 5)$
η^a	$U(0.6, 0.8)$	$U(0.6, 0.8)$	$U(0.2, 0.5)$	$U(0.2, 0.5)$
k_1	$U(0.2, 0.3)$	$U(0.2, 0.3)$	$U(0.2, 0.3)$	$U(0.2, 0.3)$
k_2	$U(0.2, 0.3)$	$U(0.3, 0.4)$	$U(0.3, 0.4)$	$U(0.8, 1)$

References

1. Tang S, Xiao Y, Wang N, Wu H. Piecewise HIV virus dynamic model with CD4+ T cell count-guided therapy: I. J. Theor. Biol., 2012; 308, 123-134. doi: 10.1016/j.jtbi.2012.05.022. PMID: 22659043
